# An evaluation of the SD Bioline HIV/syphilis duo test

**DOI:** 10.1177/0956462417717649

**Published:** 2017-06-29

**Authors:** Jeffrey Holden, Joshua Goheen, Mary Jett-Goheen, Mathilda Barnes, Yu-Hsiang Hsieh, Charlotte A Gaydos

**Affiliations:** 1 Division of Infectious Diseases, Johns Hopkins University, Baltimore, MD, USA; 2 Department of Emergency Medicine, Johns Hopkins University, Baltimore, MD, USA

**Keywords:** HIV, North America, screening, syphilis

## Abstract

Many health agencies now recommend routine HIV and syphilis testing for pregnant women and most-at-risk populations such as men who have sex with men. With the increased availability of highly sensitive, low cost rapid point-of-care tests, the ability to meet those recommendations has increased, granting wider access to quick and accurate diagnoses. Using blood specimens collected from a Baltimore City Health Department (BCHD) sexually transmitted infection clinic, we evaluated the SD Bioline HIV/Syphilis Duo, a rapid test that simultaneously detects antibodies to HIV and syphilis and has the potential to further benefit clinics and patients by reducing costs, testing complexity, and patient wait times. SD DUO HIV sensitivity and specificity, when compared to BCHD results, were 91.7 and 99.5%, respectively. SD DUO syphilis sensitivity and specificity, when compared to rapid plasma reagin, were 85.7 and 96.8%, respectively, and 69.7 and 99.7%, respectively, when compared to *Treponema pallidum* particle agglutination (TPPA). SD DUO syphilis sensitivity and specificity, when compared to a traditional screening algorithm, improved to 92.3 and 100%, respectively, and improved to 72.9 and 99.7%, respectively, when compared to a reverse screening algorithm. The HIV component of the SD DUO performed moderately well. However, results for the SD DUO syphilis component, when compared to TPPA, support the need for further testing and assessment.

## Introduction

Syphilis is easily and inexpensively treated in its primary stage, making early diagnosis a practical, cost-effective solution to reducing complications and minimizing spread.^1^ However, because no culture methods exist for syphilis, and direct detection via dark-field microscopy requires immediate examination of a lesion that may or may not be present during a patient's visit, a single, practical, and definitive test diagnosis remains elusive.^2^ Traditionally, a nontreponemal test, such as rapid plasma reagin (RPR) has been used as a screening test to detect antibodies to a nonspecific cardiolipin antigen, followed by a confirmatory treponemal test such as *Treponema pallidum* particle agglutination (TPPA) to detect antigen to *T. pallidum*. While both tests are relatively inexpensive, both require specialized equipment, technical expertise, and a significant time commitment to accurately perform; factors that can limit the ability of many facilities to provide in-house testing services, leading to the higher per test costs and treatment delays associated with off-site testing.^3^ Recently, many facilities have reversed their testing procedure for syphilis by using automated, high-throughput treponemal enzyme-linked immunoassays (EIAs) for screening to curtail personnel expenses through specimen batch testing. The procedure can reduce the rate of false positive RPR results; however, in high prevalence settings, such as urban-based sexually transmitted infection (STI) clinics, it might result in overtesting due to a higher proportion of false positives from previously treated infections (PTIs).^4,5^ It also creates the need for either a secondary treponemal test or clinical assessment based on a patient's sexual and treatment history to confirm positive results, which merely shifts costs from one operational setting to another within the same diagnostic chain.^6^

Unlike syphilis, treatment for HIV is long term and expensive. The economic burden and reduced survival rates associated with late-stage interventions make early diagnosis and treatment essential.^7^ Traditionally, HIV has been diagnosed via antibody tests: an enzyme-linked immunosorbent assay for screening and a Western blot for confirmation.^8^ As with traditional syphilis serological methods, these tests require specialized equipment, technical expertise, and a significant time commitment to accurately perform. However, in response to Centers for Disease Control and Prevention (CDC) testing guidelines and various public health funding initiatives aimed at preventing and treating HIV, rapid HIV antigen–antibody tests have become a clinical mainstay, often providing the primary diagnostic mechanism for many testing facilities.^9–11^

With many health agencies recommending or requiring routine HIV and syphilis testing for pregnant women and most-at-risk populations such as men who have sex with men (MSM), a rapid, accurate, and easy-to-use dual-component test with a low cost could substantially benefit healthcare providers and their patients by providing simultaneous screening for syphilis and HIV from a single finger stick.^12–14^ One such device is the SD BIOLINE HIV/Syphilis Duo rapid test (Standard Diagnostics, Inc., Gyeonggi-do, South Korea), a compact, qualitative, cartridge-based immunochromatographic assay, which uses finger-stick whole blood, plasma, or sera to detect antibodies to HIV-1/2 and *T. pallidum*, and delivers results in 15–20 min.

In this study, we evaluated the performance of the SD DUO by testing deidentified archival specimens collected at a Baltimore City Health Department (BCHD) STI clinic and comparing results to clinic standard of care HIV testing, RPR, TPPA, and two syphilis screening algorithms.

## Methods

Informed consent was received from all patients and the study approved by the Johns Hopkins University Institutional Review Board and the BCHD.

Blood specimens were collected between February and September 2009 from patients receiving routine care at BCHD. Blood was centrifuged to obtain serum and plasma aliquots and the serum tested on site by clinic personnel using Macro-Vue RPR Cards (Becton Dickinson BD Microbiology Systems, Sparks, MD, USA). Serum and plasma aliquots were transported to our laboratory and stored at −80°C.

SD DUO testing took place in our laboratory in August 2014 using the stored aliquots. Of 394 serum samples tested by RPR at BCHD, seven had insufficient volumes to undergo further testing and were tested using the plasma aliquot. Following the manufacturer's instructions, 10 μl of serum or plasma was added to the cartridge sample/diluent well using a micropipette, followed by three drops (∼100 μl) of diluent squeezed from the bottle included with the kit. As recommended in the package insert, the cartridges were read within 20 min to avoid false results.

TPPA testing took place in our laboratory in April 2015 using serum or plasma and the Serodia TPPA assay (Fujirebio, Tokyo, Japan) per manufacturer's package insert. Of the original 394 serum samples tested by RPR at BCHD, 384 were tested using serum and 10 were tested using plasma due to insufficient volumes of serum.

**Figure 1:**
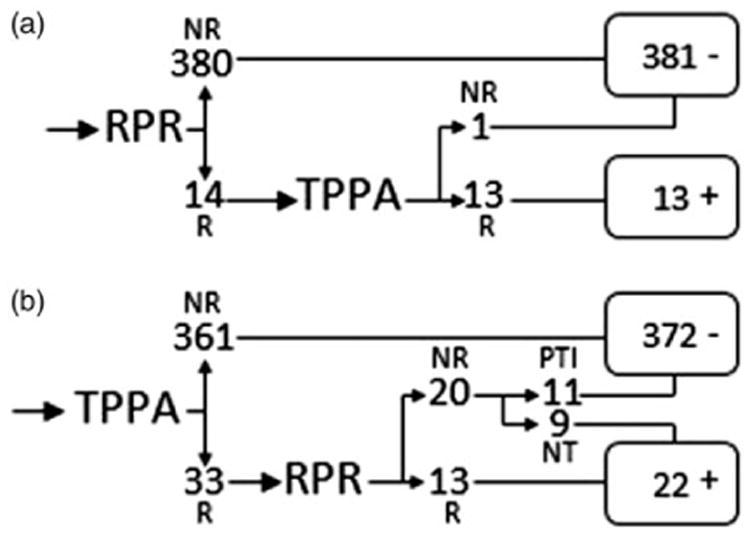
Characterization of patient infection status by simulated traditional (a) and reverse (b) algorithms (N=394). NR: nonreactive; NT: no treatment history; PTI: previously treated infection; R: reactive.

Simulated screening algorithms were used to characterize patient infection status for syphilis (Figure 1). For simulation of a traditional screening algorithm, the RPR test was designated as the nontreponemal screening test: any patients with nonreactive RPR results were deemed negative for active infection and the TPPA result excluded; otherwise the TPPA result was included in determining the patient's infection status and the patient deemed positive for syphilis if reactive. For simulation of a reverse screening algorithm, the TPPA test was designated as the treponemal screening test: any patients with nonreactive TPPA results were deemed negative for active infection and the RPR result excluded. If the TPPA result was reactive, the patient's STI history was consulted, and on evidence of a PTI, deemed negative for active infection; otherwise the patient was deemed positive for syphilis.

HIV status was ascertained by clinical personnel at the time of the blood draw using the OraQuick Advance rapid HIV-1/2 antibody test (OraSure Technologies, Bethlehem, PA, USA) or through chart review. One SD DUO HIV result was excluded due to a patient declining standard of care testing at the clinic, yielding a total of 393 evaluable specimens. The Alere Determine HIV–1/2 Ag/Ab Combo assay (Waltham, MA, USA) was used to retest specimens with apparent SD-DUO false negative and false positive HIV results.

Statistical analyses for initial and discrepant testing were performed by Stata version 14.1 (Stata Corp., College Station, TX, USA) using the exact binomial distribution to calculate confidence intervals.

## Results

Of 394 specimens tested for syphilis, 24 specimens were positive by SD DUO (6.1%), 14 specimens were reactive by RPR (3.6%), and 33 specimens were reactive by TPPA (8.4%). SD DUO sensitivity and specificity, when compared to RPR, were 85.7% (95% CI 57.2–98.2%) and 96.8% (95% CI 94.6–98.4%), respectively, with a positive predictive value (PPV) of 50.0% (95% CI 29.1–70.9%) and a negative predictive value (NPV) of 99.5% (95% CI 98.1–99.9%), respectively. SD DUO sensitivity and specificity, when compared to TPPA, were 69.7% (95% CI 51.3–84.4%) and 99.7% (CI 98.5–100%), respectively, with a PPV of 95.8 (95% CI 78.9–99.9%) and a NPV of 97.3% (95% CI 95.3–98.7%), respectively (Table 1). All apparent syphilis false negatives were from asymptomatic patients (no chancre, rash, sores, or other symptoms), four were from patients with a history of syphilis infection, and nine of ten were nonreactive via RPR, with the tenth resulting in a 1:1 RPR titer (Table 2). One apparent false positive specimen was from an asymptomatic patient with no reported history of syphilis infection (Table 2). Of 11 TPPA+/SD DUO+/RPR− specimens, four had no reported history of syphilis infection. Our simulated traditional algorithm produced 381 specimens negative for active infection and 13 specimens positive for active infection (Figure 1(a)). Our simulated reverse algorithm produced 372 specimens negative for active infection and 22 specimens positive for active infection (Figure 1(b)). When SD DUO results are compared to patient infection status as characterized by our simulated traditional and reverse algorithms, sensitivity improves to 92.3 and 72.9%, respectively (Table 3).

Of 393 specimens tested for HIV, 13 were positive by SD DUO (3.3%) and 11 were positive by health clinic testing (2.8%). SD DUO sensitivity and specificity when compared to health clinic results were 91.7% (95% CI 61.5–99.8%) and 99.5% (95% CI 98.1–99.9%), with a PPV of 84.6% (95% CI 54.6–98.1%) and a NPV of 99.7% (95% CI 98.5–100%), respectively. After discrepant testing with the tie-breaker test both HIV false positives and the HIV false negative were resolved in favor of the SD DUO test, yielding an ‘adjusted’ sensitivity and specificity of 100% (Table 4).

## Discussion

Other studies, one in the US, one in Uganda, and an international multisite study have shown results in line with the manufacturer's claims.^15–17^ All found higher sensitivities for syphilis (93.0, 100.0, and 99.7%, respectively) and comparable sensitivities for HIV (97.9, 99.1, and 100%, respectively). The disparity in sensitivities between our study and others, especially between our results for TPPA and SD DUO, both of which are treponemal tests, remains unclear; the comparatively small number of positives in our study makes it difficult to draw too many conclusions. However, as other studies of treponemal rapid tests have suggested, the positive detections missed by SD DUO in our study might be a result of lower levels of antibody titer remaining below the test's limit of detection due to testing at either the onset of syphilis antibody production or when encountering a PTI.^18,19^ Another study conducted in Peru, which found a slightly lower sensitivity for the syphilis component of the SD DUO (89.7%), noted an increase in positive results by extending the reading time from 20 to 60 min, leading them to speculate that the low intensity of the cartridge's color bands was contributing to the lower sensitivity.^20^ However, we made no attempt to read the color bands beyond the manufacturer's recommended time interval to determine if this was the case in our study.

We acknowledge several limitations. Although we were able to test discordant HIV specimens, our limited resources precluded us from testing all specimens via a gold-standard method for HIV. Also, although the manufacturer makes no sensitivity claims between whole blood, plasma, and serum, we were unable to test all specimens for syphilis utilizing the same specimen type due to insufficient specimen volumes. Whether specimen type influenced the continuity of our results was not investigated. However, it should be noted that out of seven specimens tested for syphilis by SD DUO using the plasma aliquot, three yielded apparent false negative results. The use of another treponemal test, such as FTA-ABS or EIA, to retest specimens with discordant syphilis results or a random subset of the original 394 specimens might have provided additional insight into any specimen-based cause of disparity between ours and other study's results. Also, assessment of a patient's current syphilis infection status requires an experienced clinician in physical consultation with the patient and the patient's medical history to make an accurate diagnosis. Our characterization of TPPA reactive specimens from asymptomatic patients as positives, and RPR nonreactive specimens as negatives, potentially over-or understated actual infection status. Because our study was strictly a technical comparison of multiple devices performed in a laboratory using archival specimens, no attempt at replicating an actual clinical diagnostic setting was possible. As such, our application of the traditional and reverse diagnostic algorithms, while based on patient history, failed to capture the nuanced interpretation of an experienced clinician at the time of a patient's visit and was included to highlight the complexities associated with current syphilis diagnosis.

In recent years, HIV infection rates have declined from their peak levels in developed countries. The decline is attributable in part to increased government funding, sexual health education initiatives, and effective treatment regimens.^21–23^ It can also be attributed to technological advances in sensitive diagnostic tools that have better enabled clinicians to accurately treat and monitor their patients.^24^ However, lack of funding remains a serious hurdle for many healthcare facilities struggling to provide essential services.^25,26^ Consequently, in resource-poor communities, and niche populations such as MSM and pregnant women, syphilis and HIV infection rates have increased.^27,28^ Regardless of the economic merits of traditional versus reverse algorithm syphilis screening, studies have shown that patients will not wait for an extended period at the clinic for results, and a number of patients never return for follow-up treatment once they have left the clinic.^29,30^ Thus, money saved by batch testing might be at the expense of a wider health burden due to potentially infectious patients remaining untreated.

With its price point, ease of use, and ability to rapidly detect HIV and treponemal antibodies with a high PPV and NPV, the SD DUO represents a potentially valuable reverse algorithm screening device that could well serve resource-challenged environments and communities with limited access to healthcare systems. However, our study's improved sensitivity when applying a traditional syphilis testing algorithm suggests its best use might be to confirm treponemal infections of asymptomatic patients whose treatment history is known.

## Figures and Tables

**Table 1:** Sensitivities, specificities, prevalence, and positive and negative predictive values of SD DUO compared to RPR and TPPA.

*N* = 394		RPR		TPPA	
	
		+	−	+	−
SD DUO	+	12	12	23	1
	−	2	368	10	360
		%	95% CI	%	95% CI
		
Sensitivity		85.7	57.2–98.2	69.7	51.3–84.4
Specificity		96.8	94.4–98.4	99.7	98.5–100
Prevalence		3.6	2.0–5.9	8.4	5.8–11.6
PPV		50.0	29.1–70.9	95.8	78.9–99.9
NPV		99.5	98.1–99.9	97.3	95.2–98.7

NPV: negative predictive value; PPV: positive predictive value; RPR: rapid plasma reagin; TPPA: *Treponema pallidum* particle agglutination.

**Table 2:** Characteristics of specimens with apparent false negative and false positive results for treponemal antibody from asymptomatic patients.

Specimen ID	RPR	TPPA	SD DUO	STI history	Past syphilis infection
14	−	+	−	Multiple infections	NR
28	−	+	−	Multiple infections	NR
40	−	+	−	Multiple infections	NR
44	1:1	+	−	Multiple infections	NR
63	−	+	−	Multiple infections	NR
200	−	+	−	Multiple infections	Yes
227	−	+	−	Multiple infections	Yes
248	−	+	−	Self-reported trichomoniasis	NR
301	−	+	−	Multiple infections	Yes
348	−	+	−	Syphilis	Yes
259	−	−	+	Multiple infections	NR

NR: not reported on patient questionnaire or patient chart; RPR: rapid plasma reagin; TPPA: *Treponema pallidum* particle agglutination.

**Table 3:** Sensitivities, specificities, prevalence, and positive and negative predictive values of SD DUO results compared to simulated screening algorithms.

*N* = 394		Traditional		Reverse	
	
		+	−	+	−
SD DUO	+	12	0	16	1
	−	1	381	6	371
		%	95% CI	%	95% CI
		
Sensitivity		92.3	64.0–99.8	72.9	49.8–89.3
Specificity		100	99.0–100	99.7	98.5–100
Prevalence		3.3	1.8–5.6	5.6	3.5–8.3
PPV		100	73.5–100	94.1	71.3–99.9
NPV		99.7	98.6–100	98.4	96.6–99.4

NPV: negative predictive value; PPV: positive predictive value.

**Table 4:** Sensitivities, specificities, prevalence, and positive and negative predictive values of SD DUO compared to Baltimore City Health Department's STI clinic HIV standard of care testing before and after discordant testing.

*N* = 393		BCHD			

		Before		After	
	
		+	−	+	−
SD DUO	+	11	2	13	0
	−	1	379	0	380
		%	95% CI	%	95% CI
		
Sensitivity		90.9	58.7–99.8	100	75.3–100
Specificity		99.5	98.1–99.9	100	99.0–100
Prevalence		2.8	1.4–5.0	3.31	1.8–5.6
PPV		83.3	51.6–97.9	100	75.3–100
NPV		99.7	98.6–100	100	99.0–100

BCHD: Baltimore City Health Department; NPV: negative predictive value; PPV: positive predictive value.
